# Fulminant Myocarditis With ST Elevation and Cardiogenic Shock in a SARS-CoV-2 Patient

**DOI:** 10.7759/cureus.16149

**Published:** 2021-07-03

**Authors:** Khuzema Ghafoor, Aftab Ahmed, Mubasher Abbas

**Affiliations:** 1 Internal Medicine, Atrium Health Navicent, Macon, USA; 2 Pulmonary and Critical Care Medicine, Atrium Health Navicent, Macon, USA

**Keywords:** covid 19, covid-induced myocarditis, hemodynamic shock, covid-19 and cardiomyopathy, covid-19 and heart

## Abstract

Severe acute respiratory syndrome coronavirus 2 (SARS-CoV-2) was first described in patients in Wuhan, China, who presented with flu-like symptoms. Since then, it has spread all over the world and in March 2020 it was labeled as a pandemic by the World Health Organization. Most common presentations include respiratory symptoms that vary from mild cough and shortness of breath to severe acute respiratory distress syndrome. Gastrointestinal symptoms like nausea, vomiting and diarrhea are also common. However, cardiovascular complications have not been reported widely. Patients can present with cardiac complications that include chest pain, heart failure and fulminant myocarditis, which is one of the most serious cardiac manifestations. Primary means of diagnosis are echocardiogram and cardiac magnetic imaging. Treatment is mostly supportive in case of cardiogenic shock and includes ionotropic support with or without mechanical circulatory support and mechanical ventilation. A strong suspicion is required for early diagnosis and aggressive treatment in order to reduce mortality and morbidity.

## Introduction

The first case of severe acute respiratory syndrome coronavirus 2 (SARS-CoV-2) was diagnosed in Wuhan, China, in December 2019 [1]. Since then, it has spread all over the world. Clinical presentation ranges from asymptomatic infection to severe respiratory failure requiring mechanical ventilation. Common symptoms include shortness of breath, cough, dyspnea, nausea, and vomiting [1]. However, loss of smell and taste has also been reported, and these symptoms are more common in men [2]. Chest X-ray findings typically show bilateral infiltrates with consolidation or ground-glass opacities [3-5]. Lab abnormalities include lymphopenia, elevated d-dimer levels, elevated ferritin levels, acute renal failure, and transaminitis. However, cardiac injury and elevated troponins have also been reported [1,6-8]. We present a case of a SARS-CoV-2 patient who presented with cardiogenic shock and ST elevation but was found to have SARS-CoV-2-induced myocarditis. This case adds to the current limited but significant literature of serious cardiac complications of SARS-CoV-2.

## Case presentation

A 54-year-old woman with a history of hypertension, obesity, and heart failure with preserved ejection fraction presented to the emergency room with dyspnea, nausea, and vomiting. The patient was diagnosed with SARS-CoV-2 one week before presentation at a pharmacy where she presented with mild cough and shortness of breath, and she was in self-quarantine. However, her symptoms got worse, and she decided to come to the hospital. On arrival, her blood pressure was 84/56 mm Hg, she was afebrile, her heart rate was 89 beats per minute, and her respiratory rate was 30 breaths per minute. Her chest X-ray did not show any infiltrates at the time of her presentation, and her laboratory workup showed troponin of 54 ng/ml, white cell count of 23 x 10-3/mL, Hb of 8.2 g/dl, and platelet count of 179,000. Her sodium was 158 mmol/L with acute renal failure, and her anion gap was 29. Her lactic acid was greater than 13.3 mmol/L, and d-dimer was >20.00 ug/mL. A nasopharyngeal swab came back positive for SARS-CoV-2.

**Figure 1:**
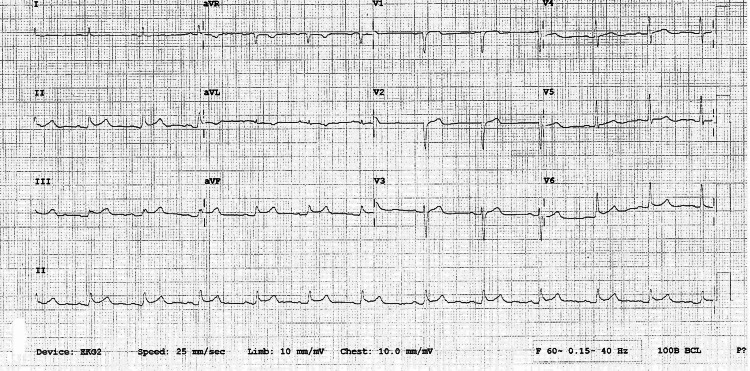
ST elevation in leads II, III and avF suggesting inferior wall ST elevation myocardial infarction.

Her EKG showed 1 mm ST elevation in the lead II, III, and aVF suggestive of inferior ST-elevation myocardial elevation (Figure 1). The patient underwent right and left heart catheterization. This demonstrated normal epicardial coronary arteries with a left ventricular ejection fraction of 10% to 15%, significantly elevated right and left cardiac filling pressures, moderate pulmonary hypertension, and severely diminished cardiac output and cardiac index of 1.0. 

While in the catheterization lab, the patient had cardiac arrest due to pulseless electrical activity after her catheterization. Cardiopulmonary resuscitation was done for 43 min to achieve a return of spontaneous circulation, and she was started on ionotropic agents and vasopressors, including norepinephrine, epinephrine, dopamine, and phenylephrine. The patient was then transferred to the intensive care unit (ICU), and she was cannulated for veno-arterial (VA) extracorporeal membrane oxygenation (ECMO) with a presumptive diagnosis of SARS-CoV-2 cardiomyopathy with myocarditis. 

**Figure 2:**
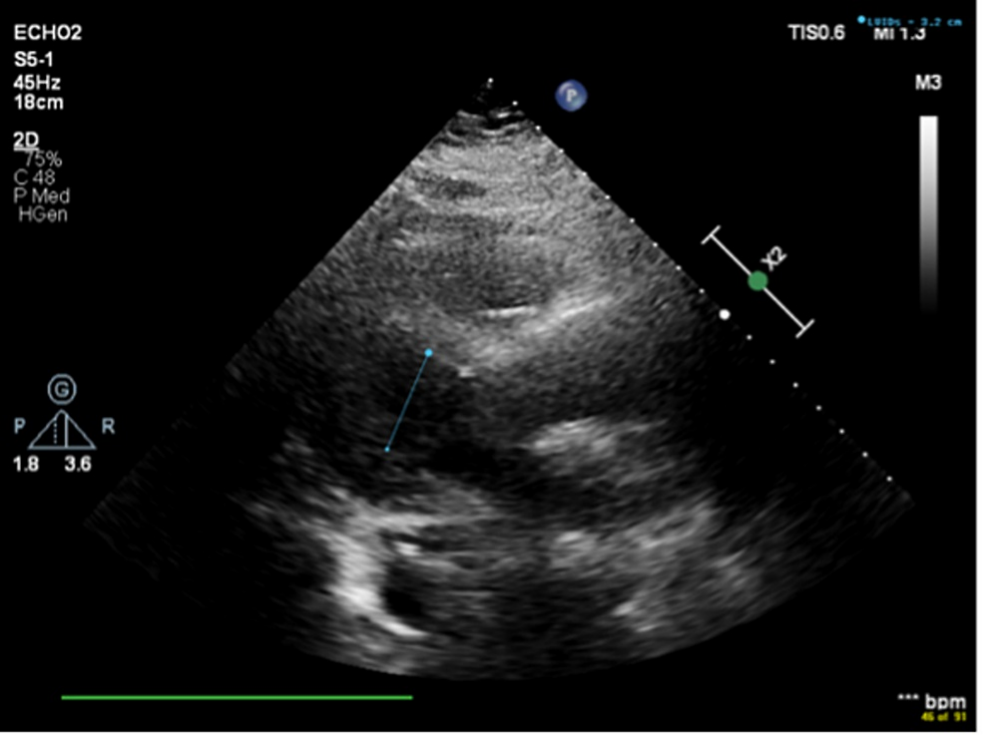
Limited 2D Echocardiogram showing Left Ventricular Internal End-Systolic Diameter (LVIDs) of 3.2 cm. (Parasternal long axis)

**Figure 3:**
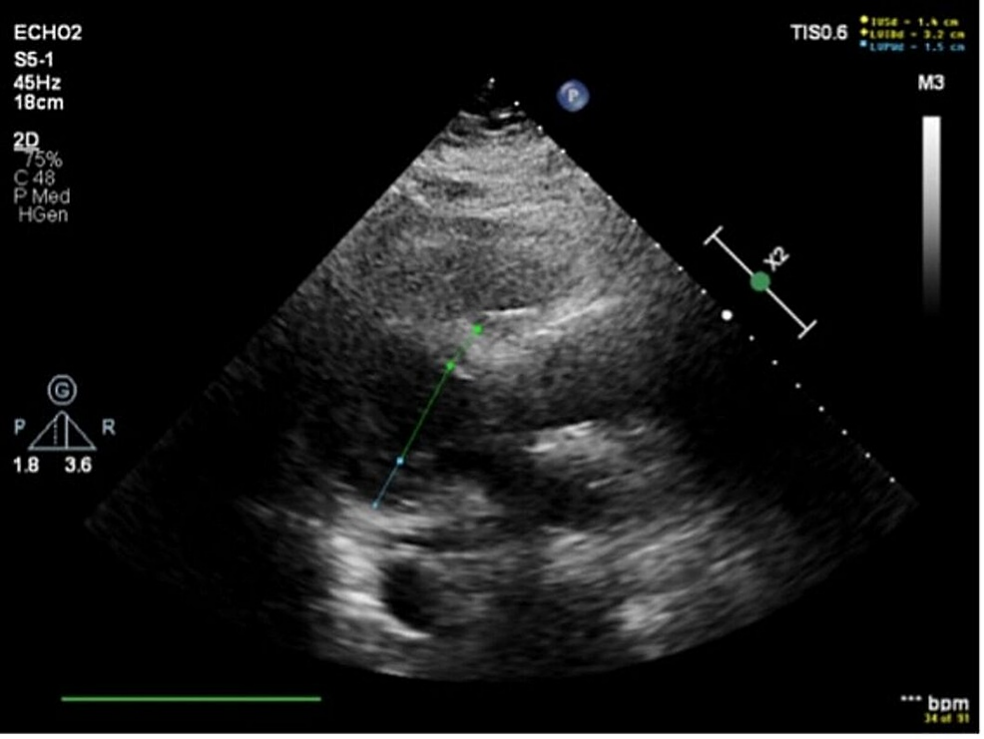
Limited 2D Echocardiogram showing Left Ventricular Internal End-Diastolic Diameter (LVIDd) of 3.2 cm. (Parasternal long axis)

In ICU, bedside echocardiogram showed severe diffuse biventricular failure and global hypokinesis with almost no motion of her heart chambers as per the echocardiogram report (Figures 2, 3, Video 1). Echocardiogram didn't show dilation of cardiac chambers, therefore suggesting acute pathology. (Figures 2, 3, Video 1). Her last echocardiogram was two years ago that showed a normal ejection fraction. Her family was educated about her clinical deterioration, and her family decided to pursue comfort care measures. 

**Video 1:** Limited Echocardiogram showing biventricular hypokinesis

## Discussion

One of the most common causes of myocarditis in the USA is viral myocarditis [9]. SARS-CoV-2-induced myocarditis is a relatively rare complication that has been reported in multiple case reports [10-14]. Symptoms can range from shortness of breath to cardiogenic shock and fulminant myocarditis, a life-threatening presentation [15]. In milder cases, patients have responded well to standard therapy of congestive heart failure, including a beta-blocker and angiotensin-converting enzyme inhibitor (ACEI) [11]. Other reported cases involving more serious presentation with biventricular failure and management involved inotropic/vasopressor support with mechanical ventilation and death due to rapid hemodynamic deterioration and cardiogenic shock [10,14]. 

There are multiple pathways by which SARS-CoV-2 infection leads to myocarditis and cardiogenic shock. Pathophysiology of viral myocarditis includes direct cell injury and T lymphocyte-mediated cytotoxicity. SARS-CoV-2 infects human cells by binding the spike protein present on its envelope with membrane protein angiotensin-converting enzyme 2 (ACE2) [16]. ACE2 is present in the respiratory tract, cardiomyocytes, and type 2 pneumocytes. The process involves cleavage of spike protein at S1/S2 and then at S2 to facilitate binding with ACE2. Transmembrane serine protease 2 (TMPRSS2) expressed on the surface of host cells plays a vital role for the spike protein to gain entry and cause host cell infection [17,18]. The excessive inflammatory response is commonly seen in patients with severe SARS-CoV-2 infection associated with the cytokine release, and it can cause acute myocardial injury [19]. There are multiple pro-inflammatory cytokines, but interleukin 6 (IL-6) plays a significant role [20]. This is supported by previous data that showed myocardial injury due to activation of T and B lymphocytes, and it caused cytokine storm with massive production of inflammatory mediators [19,21]. Another proposed mechanism based on a study of SARS-CoV-1 suggests that viral entry into cardiomyocytes triggers an innate immune response [22]. This leads to direct cytotoxicity and myocardial necrosis of varying degrees with cellular edema, hypercoagulation, endothelium, and vascular leakage [22,23]. This cascade of events can cause cardiac dysfunction due to a lack of contractility within a short period of time, and the patient presents with signs and symptoms of heart failure [22].

The 2018 Lake Louise Criteria are traditionally used to diagnose acute myocarditis, and cardiovascular magnetic resonance (CMR) has great value as a noninvasive test [24,25]. Endomyocardial biopsy is used to diagnose fulminant cases when the cause is not clear [26]. However, patients presenting with cardiogenic shock due to SARS-CoV-2 are mostly unstable to undergo either of these tests [9]. 

Therefore, diagnosis of SARS-CoV-2-induced myocarditis needs a high index of suspicion. The detailed history and physical examination and laboratory values, findings on the echocardiogram, and cardiac catheterization results help us make this diagnosis [9,27]. Patients usually present with a positive SARS-CoV-2 test with elevated NT-proBNP and cardiac troponin I. However, elevated levels are not specific for myocarditis, making it difficult to diagnose, and they are also raised in other etiologies. EKG changes are non-specific and may include ST and T wave changes as well as ST elevation. Transthoracic echocardiography (TTE) is the initial non-invasive diagnostic test for SARS-CoV-2 myocarditis and cardiogenic shock. It may show global hypokinesis, left ventricular failure, or biventricular failure [28].

Heart failure caused by SARS-CoV-2 is treated with goal-directed medical management, including beta-blockers and angiotensin-converting enzymes Inhibitors or angiotensin receptor blockers along with evidence-based management of SARS-CoV-2 infection that includes dexamethasone, remdesivir, and possibly immunoglobins for severe disease; however, the role of immunoglobins is not entirely clear [9,27,29-32]. Another trial showed baricitinib plus remdesivir was superior to remdesivir alone in accelerating improvement in patients on high flow oxygen or non-invasive ventilation, a subset that includes patients with severe myocarditis or cardiogenic shock [33]. On the contrary, convalescent plasma with high antibody titers did not reduce the risk of death in mechanically ventilated patients [34]. Treatment of cardiogenic shock and acute heart failure includes supportive care with inotropic and/or vasopressor support along with mechanical ventilation [9,10,14,27]. Extracorporeal membrane oxygenation and possibly left ventricle assist devices can also help [9,10,14,27]. 

## Conclusions

SARS-CoV-2 is a novel viral disease that was first reported in China and has since spread all over the world. It primarily affects the respiratory system and common symptoms include fever, cough, and shortness of breath; however cardiac involvement has also been noticed and can be life-threatening. Patients can present with EKG changes and hemodynamic compromise leading to rapid decompensation. This case report emphasizes the importance of high suspicion for cardiac involvement and myocarditis in SARS-CoV-2 patients who present with worsening shortness of breath along with vital instability. A prompt diagnosis is key for further management and treatment is mostly supportive involving inotropes, mechanical circulatory support, and mechanical ventilation. 
